# Large-scale quality assessment of prokaryotic genomes with metashot/prok-quality

**DOI:** 10.12688/f1000research.54418.1

**Published:** 2021-08-17

**Authors:** Davide Albanese, Claudio Donati

**Affiliations:** 1 Research and Innovation Centre, Fondazione Edmund Mach, San Michele all’Adige, TN, 38098, Italy

**Keywords:** metagenome-assembled genome, MAG, genome quality, MIMAG, dereplication, completeness, contamination, nextflow, docker

## Abstract

Metagenomic sequencing allows large-scale identification and genomic characterization. Binning is the process of recovering genomes from complex mixtures of sequence fragments (metagenome contigs) of unknown bacteria and archaeal species. Assessing the quality of genomes recovered from metagenomes requires the use of complex pipelines involving many independent steps, often difficult to reproduce and maintain. A comprehensive, automated and easy-to-use computational workflow for the quality assessment of draft prokaryotic genomes, based on container technology, would greatly improve reproducibility and reusability of published results. We present metashot/prok-quality, a container-enabled Nextflow pipeline for quality assessment and genome dereplication. The metashot/prok-quality tool produces genome quality reports that are compliant with the Minimum Information about a Metagenome-Assembled Genome (MIMAG) standard, and can run out-of-the-box on any platform that supports Nextflow, Docker or Singularity, including computing clusters or batch infrastructures in the cloud. metashot/prok-quality is part of the metashot collection of analysis pipelines. Workflow and documentation are available under GPL3 licence on GitHub.

## Introduction

Genome-resolved metagenomics is one of the most promising approaches to identify and characterize novel microbial species. Large-scale environmental and host-associated studies demonstrated how metagenomics can expand our knowledge of uncultivated prokaryotes, recovering thousands of metagenome-assembled genomes (MAGs) of new archaeal and bacterial species. ^1, 2^ For this reason, automated and reproducible methods for assessing the quality of MAGs play a critical role.

To recover MAGs, metagenomic sequence reads are first assembled into contigs using specific algorithms. ^3^ Contigs are then processed by tools like MetaBAT 2 ^4^ or VAMB ^5^ that use tetra-nucleotide frequency (TNF) profiles and abundance patterns to group sequences that are likely to belong to the same organism (binning). Binning improves the interpretability of metagenomic data, but at the same time represents (together with assembly) a significant source of error. ^6^ Manual refinement ^7^ can increase the quality of resulting MAGs, but undermines the reproducibility of the analysis and is unfeasible for large-scale studies.

The recently introduced Minimum Information about a Metagenome-Assembled Genome (MIMAG) standard ^8^ recommends a set of measures for assessing the quality of MAGs. This comprises basic assembly statistics (e.g. N50), genome *completeness,* c *ontamination* and the presence of ribosomal RNA (rRNA) and transfer RNA (tRNA) genes.

Recovering this information involves computational pipelines composed of a series of specialized tools that are often difficult to use and install. Moreover, each task can require parameters and custom scripts that are often poorly documented, making reproducibility of results challenging. Tools and standards such as Galaxy, ^9^ Nextflow ^10^ and the Common Workflow Language, ^11^ coupled with container technologies like Docker, allows researchers to circumvent these issues, providing a way to build, run and share reproducible computational workflows. ^12^

We present metashot/prok-quality, a comprehensive and easy-to-use Nextflow pipeline for assessing the quality of draft prokaryotic genomes. Metashot/prok-quality reports the quality statistics and estimates recommended by the MIMAG standard. Basic assembly statistics, completeness, both redundant and non-redundant contamination, rRNA and tRNA genes are reported in a single, comprehensive table.

## Methods

### Implementation

**Figure 1.**
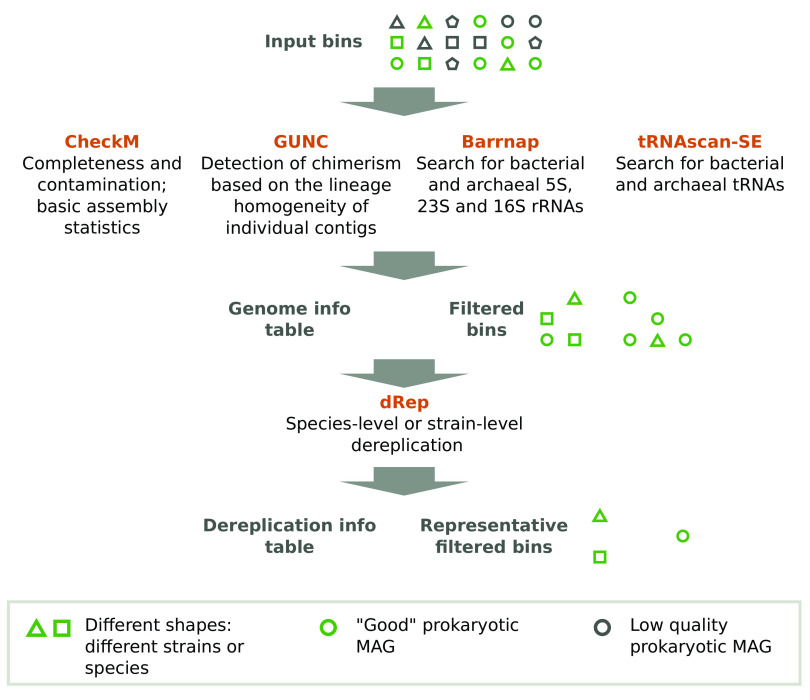
Metashot/prok-quality workflow. The workflow takes a series of genomes (input bins) in FASTA format and returns: i) a tab-separated values (TSV) file including, for each input genome, the quality information recommended by the MIMAG standard (genome info table); ii) a directory containing the bins filtered according the completeness and contamination thresholds; iii) a TSV file listing the cluster membership of each genome after the dereplication (optional) and iv) a directory containing the cluster representatives. The original outputs of each task (e.g. Barrnap’s GFF output) are also reported in dedicated folders.

Metashot/prok-quality is written using the Nextflow domain-specific language. Nextflow is a framework for building scalable scientific workflows using containers, allowing implicit parallelism on a wide range of computing systems. Reproducibility is guaranteed by versioned Docker images, which enclose software applications together with their dependencies, allowing isolation from the host environment and portability across platforms. metashot/prok-quality v1.2.0 is composed of five main modules ( Figure 1) and includes several custom scripts, designed to manipulate the output of the different tasks.

Software included in version 1.2.0:

*CheckM v1.1.2.* Several tools have been developed for the assessment of completeness and contamination of MAGs. The proposed workflow includes the widely used CheckM ^13^ which estimate these metrics using ubiquitous and lineage-specific, single-copy core genes (SCGs) catalogs. CheckM is also used to recover the basic assembly statistics.

*G* *UNC v1.0.1.* SCG-based tools like CheckM can have very low sensitivity towards contamination by fragments from unrelated organisms (non-redundant contamination). ^6^ In order to circumvent this problem, the recent GUNC ^14^ tool was added to the pipeline. GUNC quantifies the lineage homogeneity of contigs with respect to the full gene complement, accurately detecting chimerism induced by both redundant and non-redundant contamination.

*Barrnap v0.9.* The presence of 5S, 23S and 16S rRNA genes is predicted by the BAsic Rapid Ribosomal RNA Predictor ( Barrnap) using Hidden Markov models (HMM). Both bacteria and archaea databases are used.

*tRNAscan-SE v2.0.6.* tRNA genes are searched using tRNAscan-SE, ^15^ using bacteria and archaea covariance models. The number of tRNAs and tRNA isotypes found is reported.

*dRep v2.6.2.* Dereplication is a procedure that groups the input genomes according to their whole-genome similarity, using metrics such as the Average Nucleotide Identity ^16^ (ANI). Dereplication dramatically simplifies downstream analysis when the input genomes come from different sources. ^17^ In the proposed workflow, filtered genomes (genomes that pass completeness, contamination and GUNC filters) are optionally dereplicated using dRep. ^18^ For each cluster, dRep reports, as the cluster representative, the best-scoring MAG using the CheckM’s quality estimates. The score is computed using the following formula:

score = completeness − 5 × contamination + 0.5 × log(N50)

*Python3 custom scripts.* The workflow includes three Python3 custom scripts, designed to manipulate the output of the different steps. The scripts make use of NumPy, ^17^ Pandas and scikit-learn libraries.

### Operation

metashot/prok-quality v1.2.0 requires Docker and Nextflow (tested on v20.07.1). Alternatively, the Singularity container engine can be used in place of Docker. At least 70 GB of RAM is required, a limit imposed by CheckM (v1.1.2). The workflow can run in a workstation with 16 GB of RAM using the options --reduced_tree and --max_memory 16.GB.

## Use case

As mentioned above, metagenome assembly tools combine the sequence reads into larger regions called contigs. Recently, many metagenomic assembly tools have been proposed. Amongst these, metaSPAdes ^3^ and MEGAHIT ^19^ have been shown to be able to efficiently handle large-scale short read sequencing data, producing high-quality contigs. Metagenomics contigs are then processed by tools like MetaBAT 2 ^4^ in order to group sequences that are likely to belong to the same organism (binning). After binning, it is essential to assess the quality of the resulting candidate draft genomes.

In this section, we will show how to assess the quality of draft prokaryotic genomes using metashot/prok-quality. Given a series of candidate genomes in FASTA format stored in the “bins” directory, the version 1.2.0 of the workflow can be run with the following command line:
nextflow run metashot/prok-quality -r 1.2.0
\--genomes 'bins/*.fa'
\--outdir results


A series of files and directories are created in the output directory results. The main output file is “genome_info.tsv”. This TSV file contains, for each input genome, a set of quality statistics, including completeness, contamination, GUNC filter, N50, rRNA genes found, number of tRNA and tRNA types. The columns included in this file are:

• Genome: the genome filename;
• Completeness, Contamination, Strain heterogeneity: CheckM estimates;
• GUNC pass: if a genome does not pass GUNC analysis it means it is likely to be chimeric;
• Genome size (bp), ... , # predicted genes: basic genome statistics (see https://github.com/Ecogenomics/CheckM/wiki/Genome-Quality-Commands#qa);
• 5S rRNA, 23S rRNA, 16S rRNA: “Yes” if the rRNA gene was found;
• # tRNA, # tRNA types: the number of tRNA and tRNA types found, respectively.

The directory “filtered” contains the genomes (in FASTA format) filtered according to --min_completeness, --max_contamination and --gunc_filter options (see below). The TSV file “genome_info_filtered.tsv” includes the same information as “genome_info.tsv”, but for the filtered genomes only. Representative (dereplicated) genomes (default ANI threshold 0.95) are reported in the “filtered_repr” folder. The companion file “derep_info.tsv” contains the summary of the dereplication procedure, including the genome filename, the cluster ID and the representativeness. A set of secondary directories contains the original output of each tool included in the pipeline:

• checkm: contains the original CheckM's “qc” file;
• gunc: contains the original GUNC output file;
• barrnap: includes the predicted rRNA sequences for bacteria (.bac) and archaea (.arc) models in GFF and FASTA formats;
• trnascan_se: includes the predicted tRNA sequences for bacteria (.bac) and archaea (.arc) models in TSV and FASTA formats;
• drep: dRep original data tables, figures and log file.

The command options are:

Input and output

• --genomes: input genomes/bins in FASTA format (default “data/*.fa”);
• --ext: FASTA files extension, files with different extensions will be ignored (default “fa”);
• --outdir: output directory (default “results”);
• --gunc_db: the GUNC database. If “none” the database will be automatically downloaded and will be placed the output folder (gunc_db directory) (default “none”);

CheckM

• --reduced_tree: reduce the memory requirements to approximately 14 GB, set --max_memory to 16.GB (default false);
• --checkm_batch_size: run CheckM on “checkm_batch_size” genomes at once in order to avoid memory issues, see https://github.com/Ecogenomics/CheckM/issues/118 (default 1000);

GUNC

• --gunc_batch_size: run GUNC on “gunc_batch_size” genomes at once (default 100);

Filtering

• --min_completeness: discard sequences with less than “min_completeness” % completeness (default 50);
• --max_contamination: discard sequences with more than “max_contamination” % contamination (default 10);
• --gunc_filter: if true, discard genomes that do not pass the GUNC filter (default false);

Dereplication

• --skip_dereplication: skip the dereplication step (default false);
• --ani_thr: ANI threshold for dereplication (> 0.90) (default 0.95);
• --min_overlap: minimum required overlap in the alignment between genomes to compute ANI (default 0.30);

Resource limits

• --max_cpus: maximum number of CPUs for each process (default 8);
• --max_memory: maximum memory for each process (default 70.GB);
• --max_time: maximum time for each process (default 96.h).

## Software availability

Source code available from: https://github.com/metashot/prok-quality

Archived source code at time of publication: http://doi.org/10.5281/zenodo.4475355. ^20^

License: GPL-3.0

Docker image definitions available from: https://github.com/metashot/docker

## Data availability

### Underlying data

Zenodo: metashot/prok-quality v1.2.0 with test data, v1.2.0, http://doi.org/10.5281/zenodo.4475355. ^3^

This project contains test data and workflow documentation.

Data are available under the terms of GNU General Public License version 3 (GPL-3).

### Extended data

Docker Hub: metashot docker images, https://hub.docker.com/u/metashot

This registry contains the pre-built Docker images 

GitHub: metashot/docker, https://github.com/metashot/docker

This project contains Docker image definitions.
