# Supplementary material for: Large-scale quality assessment of prokaryotic genomes with metashot/prok-quality
Source: F1000Res. 2021 Aug 17;10:822. [Version 1] doi: 10.12688/f1000research.54418.1 (PMC8804904; doi:10.12688/f1000research.54418.1)
Supplement: Peer review history

# Peer review: Reviewer response for version 1

**DOI:** 10.5256/f1000research.57902.r100040

**Published:** 2022-01-31

**Authors:** Denis Baurain

**Affiliations:** 1 InBioS - PhytoSYSTEMS, Eukaryotic Phylogenomics, University of Liège, Liège, Belgium

This tool is useful and works as advertised by the authors. The manuscript reads well and I only have very minor comments.

**Functionality:**

 I tested metashot/prok-quality on a HPC facility running the SLURM grid engine. The authors' instructions were nearly enough to make it work on my first attempt. However, getting right the Singularity options was a little trickier than expected. In this regard, Docker is easier to use but generally not available in HPC environments. I contacted the authors by email (posing as a regular user) and they replied immediately, confirming that the reported issue was on my side and not a bug in their package. Unfortunately, I had to attend more pressing matters and only got back to the issue recently, hence this delayed review report. I apologize for this. For the record, here are the lines required to run the tool successfully on my system:

 # in prok-quality/nextflow.config

 singularity.enabled = true

 singularity.cacheDir = "$PWD"

 singularity.autoMounts = false

 singularity.runOptions = "-B /path-to-user-home/prok-quality-1.2.0_with_test_data/ -B /tmpscratch/username"

 The interface of metashot/prok-quality is straightforward and well designed, with attention to details, such as the possibility to deal with limited RAM ( --reduced_tree option for CheckM) and to economize resources ( --gunc_db option to avoid downloading the GUNC database multiple times). I only have a minor complaint: the --genomes option expects input filenames but if these do not match the --ext option (e.g., .fasta instead of .fa), infiles are silently ignored. In my view, the --ext option would make more sense with a --genomes option expecting an input directory. Indeed, when infiles are specified, there is no real need for filtering.

 The tool's outputs are easy to understand and well documented. Testing it with default thresholds on a chimerical bacterial genome (Cornet and Baurain 2022, Genome Biology, in press) indeed flags it as contaminated and excludes it from the downstream dereplication step. In contrast, the two corresponding clean genomes undergo through all the steps of the pipeline, as expected.

**Manuscript:**

- **Abstract:**
  - Eukaryotic sequences can be found in metagenomic samples. These are more difficult to deal with, but should be mentioned in the second sentence.
  - abstract: "... platform that supports Nextflow, Docker or Singularity" should read "... platform that supports Nextflow and either Docker or Singularity".
- **Introduction: **I would suggest adding a small paragraph about the need to use multiple tools when assessing genomic contaminations. This paragraph could include (and slightly expand) the rationale for GUNC (presently located in the Methods). For some ideas, see Lupo *et al. *(2021 ^1^).
- **Methods:**
  - Some sentences are either too much or too little, for example those about the rationale for dRep. I think that one more sentence would help and it would be better to move the whole idea of dereplication at the end of the Introduction. Indeed, Methods should not explain concepts.
  - Figure 1: The figure is nice. If I had to quibble, I would say that "filtered bins" can be misleading: here, the authors mean "bins satisfying their criteria of completeness and contamination" whereas one could imagine that they mean "bins cleaned up from contaminating sequences" (i.e., bins are passed "as is" and are not modified by the pipeline). Moreover, showing on the figure the parameters controlling the thresholds would help the reader to realize that they can be user-specified. Finally, a word is missing in the legend: "according the completeness.." should read "according to the completeness..." 
  - The score formula should specify if completeness and contamination metrics are computed in percentage or not. Moreover, it might be interesting to make it user-tweakable (only a suggestion).
- **Use case:** I am not sure about the position of the backslash chars in the code snippet. I would have put them at end of lines.

Are the conclusions about the tool and its performance adequately supported by the findings presented in the article?

Yes

Is the rationale for developing the new software tool clearly explained?

Yes

Is the description of the software tool technically sound?

Yes

Are sufficient details of the code, methods and analysis (if applicable) provided to allow replication of the software development and its use by others?

Yes

Is sufficient information provided to allow interpretation of the expected output datasets and any results generated using the tool?

Yes

Reviewer Expertise:

bioinformatics, including genomic contaminations

I confirm that I have read this submission and believe that I have an appropriate level of expertise to confirm that it is of an acceptable scientific standard.

## References

[1] : Contamination in Reference Sequence Databases: Time for Divide-and-Rule Tactics. *Front Microbiol* .2021;12: 10.3389/fmicb.2021.755101 755101 10.3389/fmicb.2021.755101 34745061PMC8570097

# Peer review: Reviewer response for version 1

**DOI:** 10.5256/f1000research.57902.r100039

**Published:** 2021-11-24

**Authors:** Thomas S. B. Schmidt, Anthony Fullam, Askarbek Orakov

**Affiliations:** 1 Structural and Computational Biology Unit, European Molecular Biology Laboratory, Heidelberg, Germany

The authors present ‘prok-quality’, a one-stop-shop Nextflow workflow for prokaryotic genome quality assessment, wrapping several state-of-the-art tools into a user-friendly pipeline. prok-quality fills two relevant niches: it provides an easy to install, easy to use interface to relevant bioinformatics tools for non-expert users; and a portable, scalable workflow to process datasets of increasing sizes.

 The code is available on github. We were able to download, install and use the workflow as advertised. We have only very minor comments, to be addressed at the authors’ discretion:

  

- The authors restrict their discussion to metagenome-assembled genomes. However, there is no inherent reason why the prok-quality workflow shouldn’t be used for reference genomes or isolate sequencing as well, and this may be worth emphasizing. In practice, many users work on large integrated datasets of MAGs and reference genomes, where fast, consistent quality control and clustering using a workflow such as prok-quality is highly relevant.
- In the bigger picture of an entire genome-resolved metagenomics workflow, from raw reads to biological analyses, prok-quality covers a reasonable chunk: the quality control and ‘dereplication’ (clustering) of genomes. That way, it can be used as a module, independently of upstream (assembly & binning) and downstream (annotation, analysis) tool choices. However, in our view, prok-quality would benefit from the addition of a taxonomic classifier, e.g. GTDB-tk. Taxonomic information would fit in very well with the reported quality metrics; but in particular, the workflow could (optionally) provide consensus taxonomies for dRep 95% ANI clusters which would greatly add value for non-expert users.
- Minor: the GUNC preprint has in the meantime been peer reviewed and published (Orakov *et al. *(2021 ^1^)).
- Minor: An option to switch from running with docker to running with singularity (e.g. '-profile singularity' with config profiles) would greatly enhance the ease of using this workflow as some clusters prohibit use of docker.

Are the conclusions about the tool and its performance adequately supported by the findings presented in the article?

Yes

Is the rationale for developing the new software tool clearly explained?

Yes

Is the description of the software tool technically sound?

Yes

Are sufficient details of the code, methods and analysis (if applicable) provided to allow replication of the software development and its use by others?

Yes

Is sufficient information provided to allow interpretation of the expected output datasets and any results generated using the tool?

Yes

Reviewer Expertise:

metagenomics; microbiome

We confirm that we have read this submission and believe that we have an appropriate level of expertise to confirm that it is of an acceptable scientific standard.

## References

[1] : GUNC: detection of chimerism and contamination in prokaryotic genomes. *Genome Biology* .2021;22(1) : 10.1186/s13059-021-02393-0 10.1186/s13059-021-02393-0 34120611PMC8201837
